# Water-Soluble and -Insoluble Polymers and Biopolymers for Biomedical, Environmental, and Biological Applications

**DOI:** 10.3390/polym14122386

**Published:** 2022-06-13

**Authors:** Florian J. Stadler, Alberto García-Peñas

**Affiliations:** 1 College of Materials Science and Engineering, Shenzhen Key Laboratory of Polymer Science and Technology, Guangdong Research Center for Interfacial Engineering of Functional Materials, Nanshan District Key Laboratory for Biopolymers and Safety Evaluation, Shenzhen University, Shenzhen 518055, China; 2 Departamento de Ciencia e Ingeniería de Materiales e Ingeniería Química (IAAB), Universidad Carlos III de Madrid, 28911 Leganés, Spain

In this Special Issue, several papers dedicated to biomedical, environmental, and biological applications have been assembled, representing different aspects of the field. The works submitted to this Special Issue covered water-soluble biopolymers (shellac-coated and epoxy-coated microcapsules and poly-N-isopropylacrylamide-based polymers) as well as water-insoluble ones (poly(lactic acid) and chitosan). Further, synthetic but biodegradable polymers (poly(butyl cyanoacrylate) and poly(vinyl alcohol)) have been also covered.

The work of Yan et al. [1] focuses on the synthesis of microcapsules derived from melamine with epoxy or shellac coatings to cover lime (also called linden) tree wood. These microcapsules protect the wood mechanically by improving surface hardness. Furthermore, the coatings introduce self-healing to cracks in the wood, supposedly by bridging cracks and “gluing” them together.

Beletti et al. [2] produced poly(lactic acid) dispersions via an emulsion method, which is stable under refrigerator conditions for a long time. The size of the nanoparticles can be precisely tuned with a very homogeneous size distribution by choice of the synthesis composition—variation in solvent and surfactant concentrations—addition of starch, and mixing protocol. The hydrophilic–hydrophobic balance can be tuned by the amount of surfactant in the emulsion. The advantage of this approach is that castable films can be produced that are biodegradable.

Moral-Zamorano et al. [3] synthesized copolymers based on N-isopropylacrylamide (NIPAM) and dopamine methacrylamide (DMA)—called NIDO—and further functionalized them with the organometallic complex bis(cyclopentadienyl)titanium (IV) dichloride. This work is in a line of research investigations on NIDO and related polymers with respect to its physicochemical behavior [4,5,6,7,8,9] and for various applications, especially in the biomedical field [10]. The paper of Moral-Zamorano et al. [3] determined the effect of the loading of the organometallic complex on thermoresponsivity. While increasing the loading of the organometallic complex decreases the lower critical solution temperature somewhat, it leads to a very sharp change in the behavior, which only spans a transition temperature range of ca. 8 K, while for a normal unmodified NIDO, the transition spans ca. 25 K. Furthermore, this special polymer could also be used for its interactions with DNA.

Chitosan is one of the intensively researched biopolymers derived from chitin, the main structural polyamonisaccharide of insects, arachnids, and fungi. In recent years, chitin has attracted a tremendous amount of attention for biomedical [11,12], sustainable [13], and environmental applications [14]. Maliki et al. [15] summarized this development in their mini-review with respect to giving a short overview of the different sustainable development paths of this highly important biopolymer.

Keller et al. [16] prepared tailored molecular weights for poly(butyl cyanoacrylate) (PBCA) through an anionic polymerization process. For that purpose, the authors used the postulated depolymerization–repolymerization process (DPRP) in the literature, confirming its use for obtaining custom PBCA. Furthermore, it was observed that the end-capping of the PBCA chain banned the monomer release.

Alonso-López et al. [17] studied the biodegradation of poly(vinyl alcohol)-based materials in the marine environment. This is an essential task as maritime pollution by fishing nets, microplastic and other trash has affected every single coast on planet earth, including Antarctica, and has been found in almost every oceanic sediment, including those in the Mariana trench. For this reason, it would be highly desirable to replace some of the materials used as packing with new materials that degrade rapidly under marine conditions instead of creating continued pollution of the oceans for at least the next few centuries (if humanity would stop polluting them today). Alonso-López et al.’s work improved the biodegradability of poly(vinyl alcohol) by blending it with glycerol, which increased the biodegradability, albeit not enough to be a genuinely biodegradable material [17].

These six papers in this Special Issue, although not covering the full range of the vast biopolymer field, give a good overview of some important topics that are required for a sustainable future. Clearly, continuing to use enormous amounts of non-degradable synthetic polymers will spell ecological disaster, for which using biopolymers is part of the solution for preventing it. Due to their biocompatible nature, biopolymers will be suitable for in vivo applications, which have been partially investigated in this Special Issue. However, their properties are significantly more complex than traditional synthetic polymers, making an in-depth understanding of biopolymer physics necessary. In the future, we wish that the special properties of biopolymers can be used to reduce humanity’s inflicted ecological damage and improve society, especially with respect to supporting the healthcare of an aging population.
